# Supplementary material for: Genome-wide association study identifies 16 genomic regions associated with circulating cytokines at birth
Source: PLoS Genet. 2020 Nov 23;16(11):e1009163. doi: 10.1371/journal.pgen.1009163 (PMC7721185; doi:10.1371/journal.pgen.1009163)
Supplement: S1 Text — (PDF) [file pgen.1009163.s001.pdf]

Supplementary Text

Genome-wide Association study identifies 16 genomic regions associated with circulating cytokines at birth

Wang *et al.*

S1 Text

Replication of previous associations

We re-identified the well-known association of rs3091244 (**S1 Fig**) with CRP level[1], the HLA region with IgA[2](**S14 Fig**), the IL18 gene with IL18[3, 4](**S15 Fig**), the CCR3 and ACKR1/2 genes with MCP1[3, 5, 6](**S19-22 Figs**), the ACKR2 and CCL17 gene with TARC[3, 7](**S25-28 Figs**), as well as the 6q21.1 and 9p24.2 regions with VEGFA levels[8](**S29 and S30 Figs**). We also replicated associations reported by Ahola-Olli et al.[3] for markers, MCP1, IL18 and VEGFA, investigated here (**S10 Table**).

Co-localize associated SNPs with expression QTL.

The association statistics (p values) was used to find colocalized eQTLs from the GTEx project[9] (release V6p) dataset was downloaded from the public database. The online program SherLock were used[10]. SNPs with association p value < $5x10^{-8}$ and colocalization log_{10} Bayesian Factor (LBF)>4 were shown in the sheet 5 of Table S2-8 for CRP, EPO, IL18, MCP1, S100B, TARC and VEGFA levels. We also annotated SNPs to eQTLs using HaploReg v4.1[11]. The results, if such co-expression exists, are listed in sheet 6 of the **S2-8 Tables**.

Enrichment of association in epigenomic features

We investigated the enrichment of genetic associations of each inflammation marker separately with DNAse hypersensitive site hotspots, chromatin segmentation states and histone modification marks using the program at https://www.ebi.ac.uk/birney-srv/GARFIELD/. We observed enrichment in blood tissues for most markers but there is no clear pattern for a particular feature (**S34-36 Figs**). After Bonferoni correction, we did not obtain significant enrichment.

1. Ridker PM, Pare G, Parker A, Zee RY, Danik JS, Buring JE, et al. Loci related to metabolic-syndrome pathways including LEPR,HNF1A, IL6R, and GCKR associate with plasma C-reactive protein: the Women's Genome Health Study. American journal of human genetics. 2008;82(5):1185-92. Epub 2008/04/29. doi: 10.1016/j.ajhg.2008.03.015. PubMed PMID: 18439548; PubMed Central PMCID: PMCPMC2427311.
2. Kiryluk K, Li Y, Scolari F, Sanna-Cherchi S, Choi M, Verbitsky M, et al. Discovery of new risk loci for IgA nephropathy implicates genes involved in immunity against intestinal

pathogens. Nat Genet. 2014;46(11):1187-96. doi: 10.1038/ng.3118. PubMed PMID: 25305756; PubMed Central PMCID: PMC4213311.
3. Ahola-Olli AV, Wurtz P, Havulinna AS, Aalto K, Pitkanen N, Lehtimaki T, et al. Genome-wide Association Study Identifies 27 Loci Influencing Concentrations of Circulating Cytokines and Growth Factors. American journal of human genetics. 2017;100(1):40-50. Epub 2016/12/19. doi: 10.1016/j.ajhg.2016.11.007. PubMed PMID: 27989323; PubMed Central PMCID: PMCPMC5223028.
4. He M, Cornelis MC, Kraft P, van Dam RM, Sun Q, Laurie CC, et al. Genome-wide association study identifies variants at the IL18-BCO2 locus associated with interleukin-18 levels. Arteriosclerosis, thrombosis, and vascular biology. 2010;30(4):885-90. Epub 2010/02/13. doi: 10.1161/atvbaha.109.199422. PubMed PMID: 20150558; PubMed Central PMCID: PMCPMC2841960.
5. Voruganti VS, Laston S, Haack K, Mehta NR, Smith CW, Cole SA, et al. Genome-wide association replicates the association of Duffy antigen receptor for chemokines (DARC) polymorphisms with serum monocyte chemoattractant protein-1 (MCP-1) levels in Hispanic children. Cytokine. 2012;60(3):634-8. doi: 10.1016/j.cyto.2012.08.029. PubMed PMID: 23017229; PubMed Central PMCID: PMC3501981.
6. Comuzzie AG, Cole SA, Laston SL, Voruganti VS, Haack K, Gibbs RA, et al. Novel genetic loci identified for the pathophysiology of childhood obesity in the Hispanic population. PloS one. 2012;7(12):e51954. Epub 2012/12/20. doi: 10.1371/journal.pone.0051954. PubMed PMID: 23251661; PubMed Central PMCID: PMCPMC3522587.
7. Suhre K, Arnold M, Bhagwat AM, Cotton RJ, Engelke R, Raffler J, et al. Connecting genetic risk to disease end points through the human blood plasma proteome. Nat Commun. 2017;8:14357. Epub 2017/02/28. doi: 10.1038/ncomms14357. PubMed PMID: 28240269; PubMed Central PMCID: PMCPMC5333359.
8. Choi SH, Ruggiero D, Sorice R, Song C, Nutile T, Vernon Smith A, et al. Six Novel Loci Associated with Circulating VEGF Levels Identified by a Meta-analysis of Genome-Wide Association Studies. PLoS Genet. 2016;12(2):e1005874. Epub 2016/02/26. doi: 10.1371/journal.pgen.1005874. PubMed PMID: 26910538; PubMed Central PMCID: PMCPMC4766012.
9. Consortium G. Human genomics. The Genotype-Tissue Expression (GTEx) pilot analysis: multitissue gene regulation in humans. Science. 2015;348(6235):648-60. Epub 2015/05/09. doi: 10.1126/science.1262110. PubMed PMID: 25954001; PubMed Central PMCID: PMCPMC4547484.
10. He X, Fuller CK, Song Y, Meng Q, Zhang B, Yang X, et al. Sherlock: detecting gene-disease associations by matching patterns of expression QTL and GWAS. American journal of human genetics. 2013;92(5):667-80. Epub 2013/05/07. doi: 10.1016/j.ajhg.2013.03.022. PubMed PMID: 23643380; PubMed Central PMCID: PMCPMC3644637.
11. Ward LD, Kellis M. HaploReg v4: systematic mining of putative causal variants, cell types, regulators and target genes for human complex traits and disease. Nucleic Acids Res. 2016;44(Database issue):D877-81. doi: 10.1093/nar/gkv1340. PubMed PMID: 26657631; PubMed Central PMCID: PMC4702929.
