# Supplementary material for: Genome-wide association study identifies 16 genomic regions associated with circulating cytokines at birth
Source: PLoS Genet. 2020 Nov 23;16(11):e1009163. doi: 10.1371/journal.pgen.1009163 (PMC7721185; doi:10.1371/journal.pgen.1009163)
Supplement: S1 Table — (PDF) [file pgen.1009163.s002.pdf]

S1 Table. Description of samples

Note, there are subjects having more than one diagnosis. Thus, the total number is the true total sample size without multiple diagnoses. The age distribution for discovery sample is (years at 2012): minimum=7, maximum=32, median=22, mean=21, and, for replication sample: minimum=7, maximum=16, median=7, mean=7.

Discovery				Replication				
diagnosis		SEX		Diagnosis		SEX		
		Male	female			male	female	
SCZ	1,926	2777	2126	SCZ	0	444	118	
BIP	703			BIP	0			
ASD	1,182			ASD	325			
ADHD	1,336			ADHD	283			
AFF	2,179			AFF	10			
ANO	103			ANO	4			
control	3,268	1741	1,674	control	574	294	285	
	8,318	4,518	3800		1141	738	403	Total
