# Supplementary figures and images for: Genome-wide association study identifies 16 genomic regions associated with circulating cytokines at birth
Source: PLoS Genet. 2020 Nov 23;16(11):e1009163. doi: 10.1371/journal.pgen.1009163 (PMC7721185; doi:10.1371/journal.pgen.1009163)

S1 Fig. The Manhattan & qq plots for BDNF level

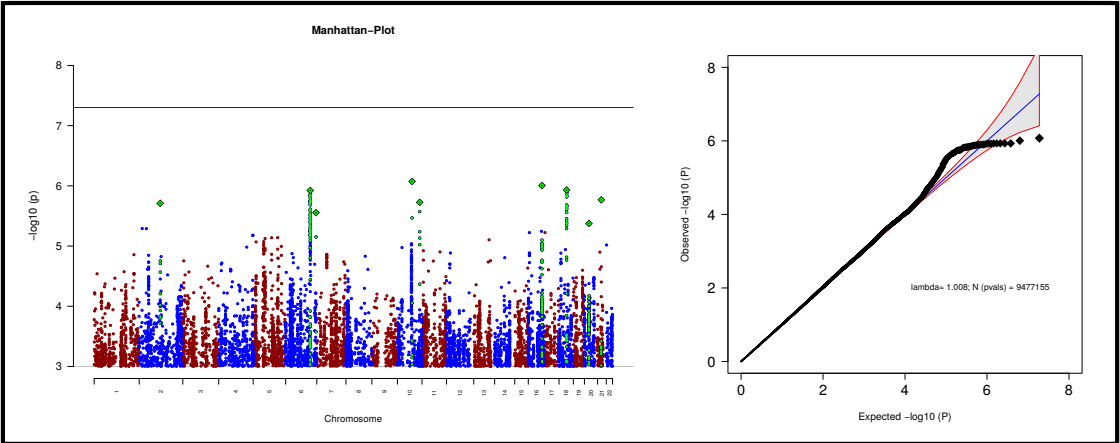

Supplement: S1 Fig — (PDF) [file pgen.1009163.s012.pdf]

S2 Fig. The Manhattan & qq plots for IL8 level

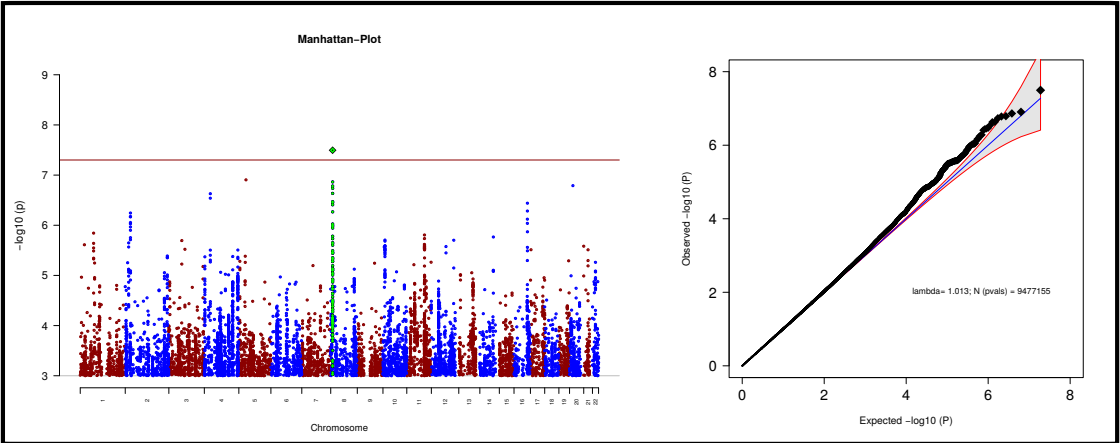

Supplement: S2 Fig — (PDF) [file pgen.1009163.s013.pdf]

S3 Fig. The Manhattan & qq plots for CRP level

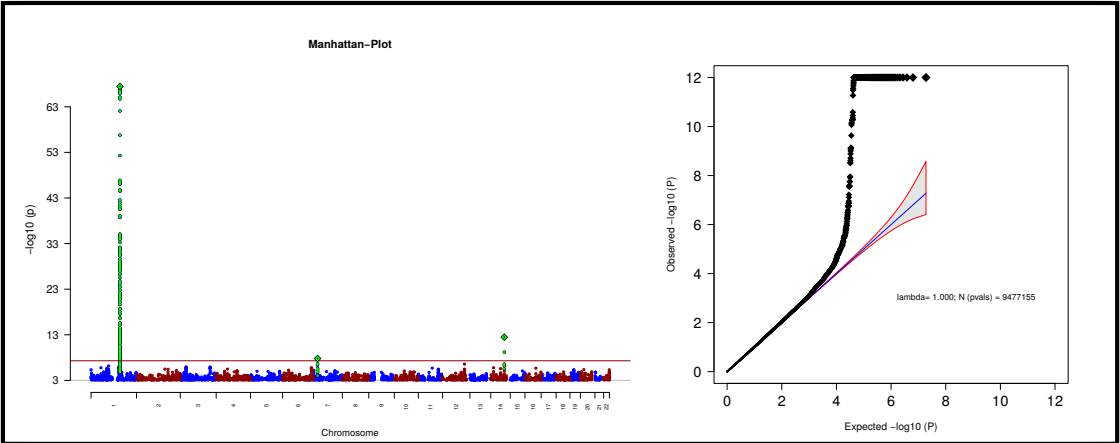

Supplement: S3 Fig — (PDF) [file pgen.1009163.s014.pdf]

S4 Fig. The Manhattan & qq plots for EPO level

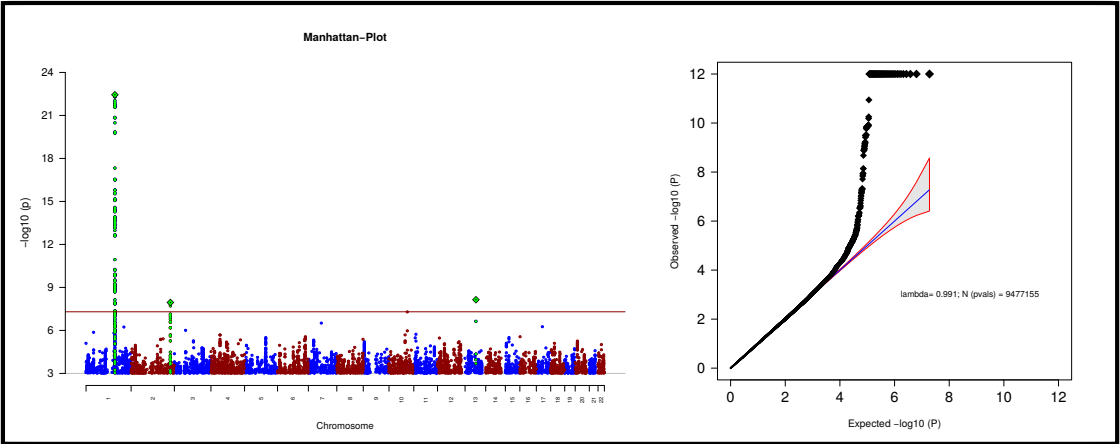

Supplement: S4 Fig — (PDF) [file pgen.1009163.s015.pdf]

S5 Fig. The Manhattan & qq plots for IgA level

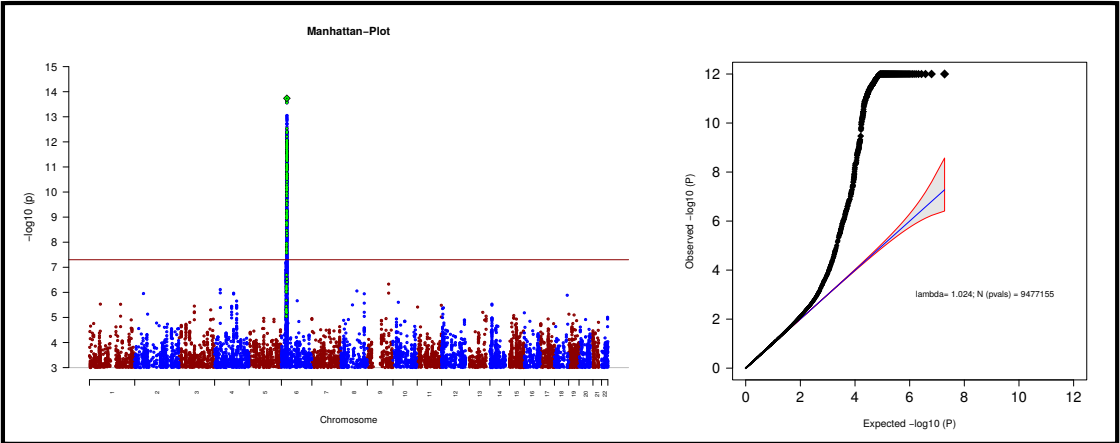

Supplement: S5 Fig — (PDF) [file pgen.1009163.s016.pdf]

S6 Fig. The Manhattan & qq plots for IL18 level

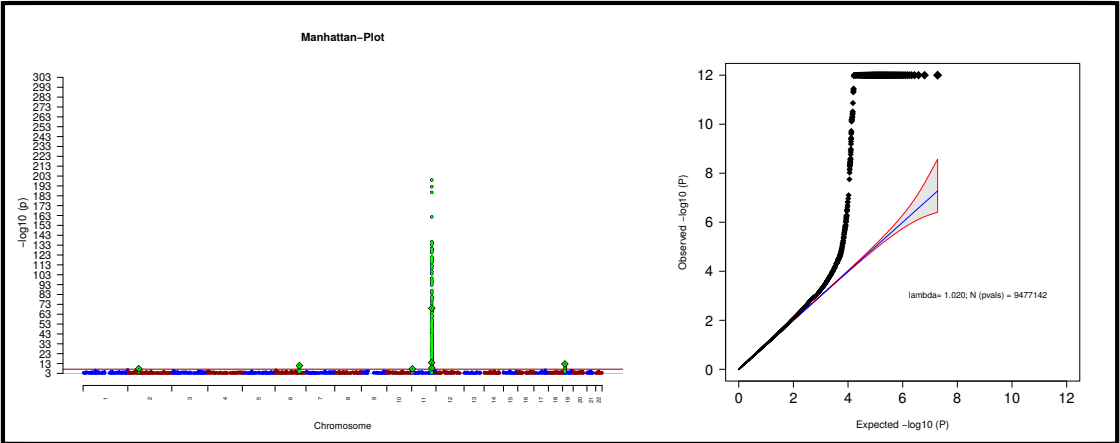

Supplement: S6 Fig — (PDF) [file pgen.1009163.s017.pdf]

S7 Fig. The Manhattan & qq plots for MCP1 level

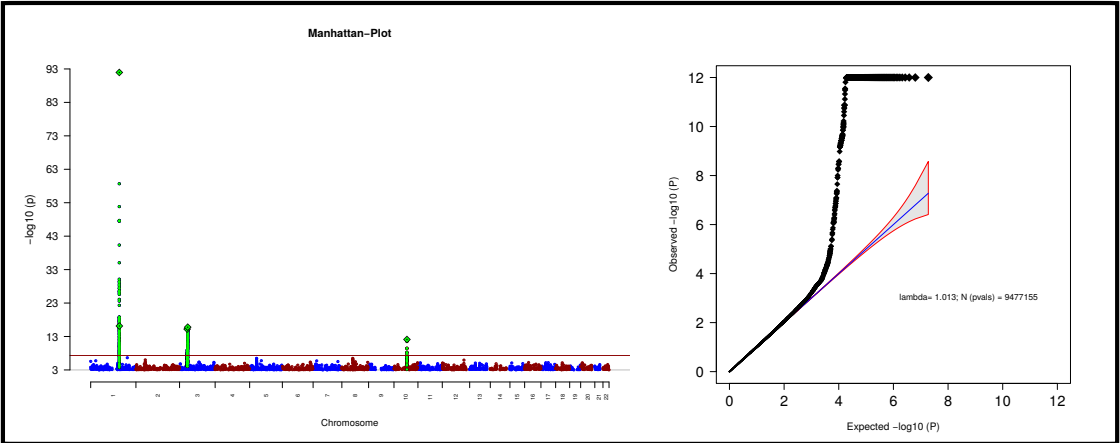

Supplement: S7 Fig — (PDF) [file pgen.1009163.s018.pdf]

S8 Fig. The Manhattan & qq plots for S100B level

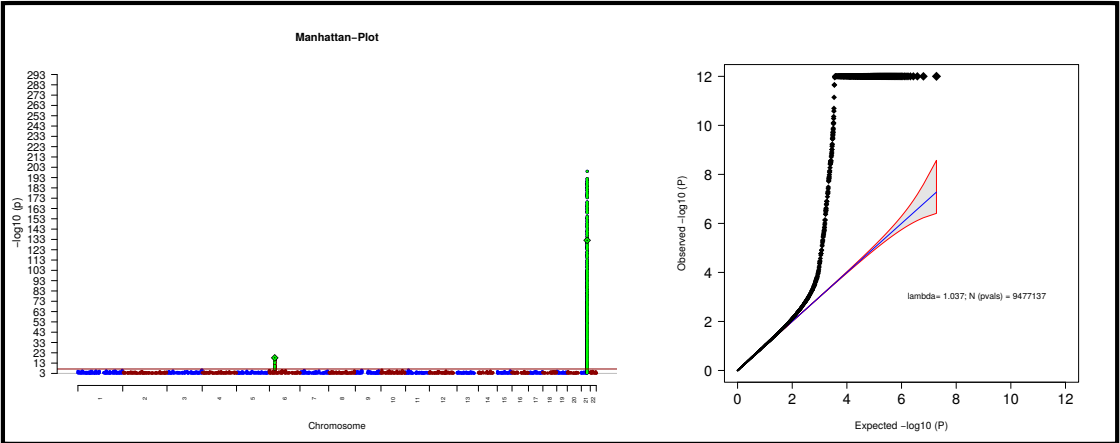

Supplement: S8 Fig — (PDF) [file pgen.1009163.s019.pdf]

S9 Fig. The Manhattan & qq plots for TARC level

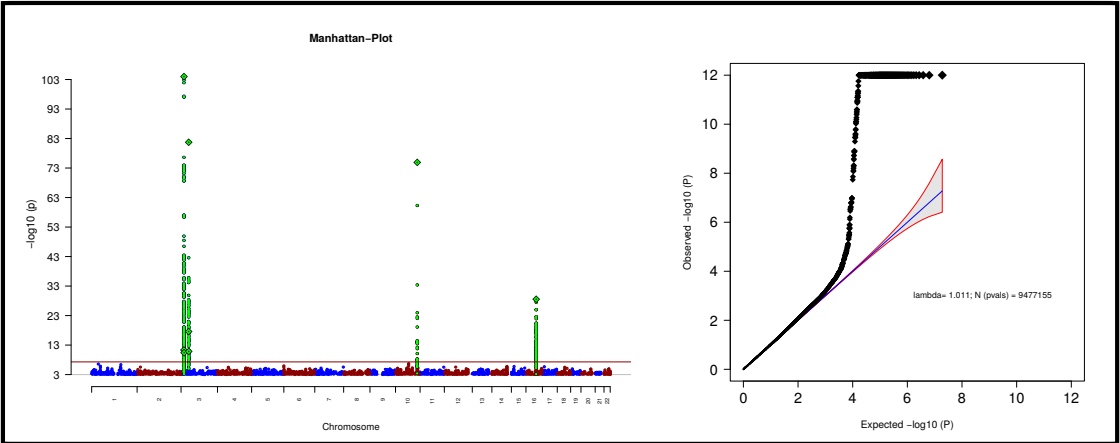

Supplement: S9 Fig — (PDF) [file pgen.1009163.s020.pdf]

S10 Fig. The Manhattan & qq plots for VEGFA level

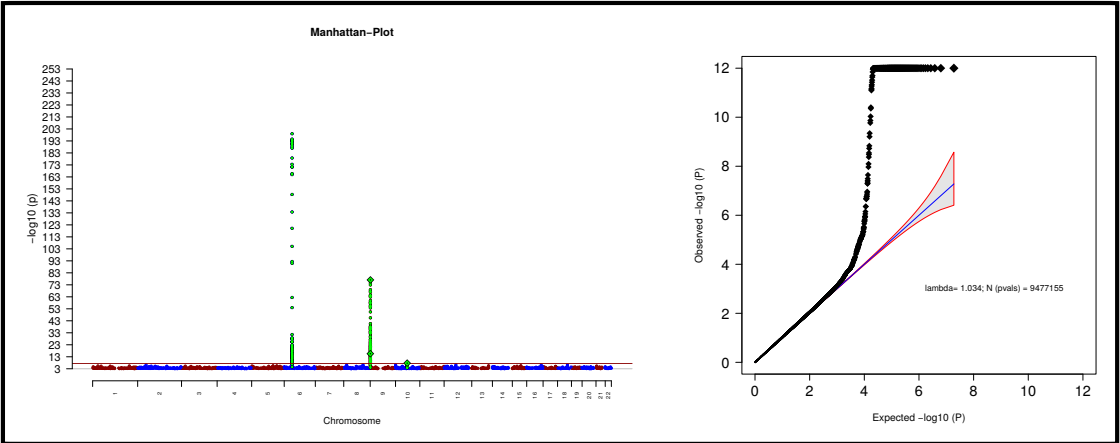

Supplement: S10 Fig — (PDF) [file pgen.1009163.s021.pdf]
