# Supplementary material for: Genome-wide association study identifies 16 genomic regions associated with circulating cytokines at birth
Source: PLoS Genet. 2020 Nov 23;16(11):e1009163. doi: 10.1371/journal.pgen.1009163 (PMC7721185; doi:10.1371/journal.pgen.1009163)
Supplement: S27 Fig — (PDF) [file pgen.1009163.s038.pdf]

S27 Fig. Region plot of TARC- rs10886430 association

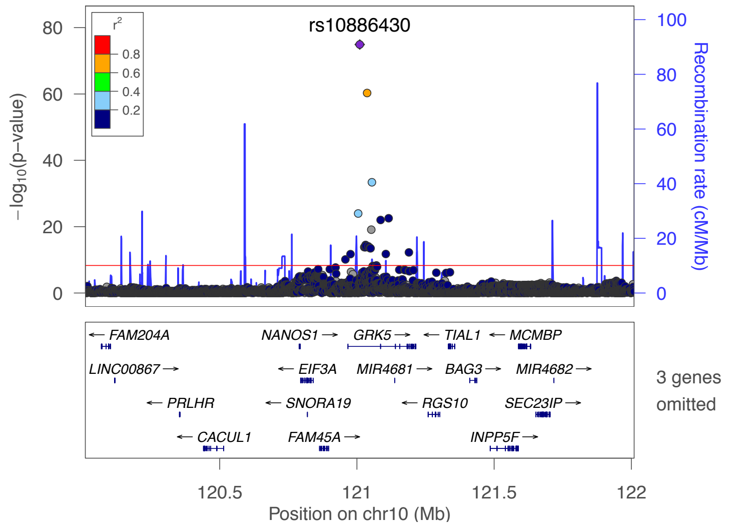

The negative common logarithm of association p values are shown on y axis; The genomic position (on the hg 19 build) is show on x axis. The symbols of genes in the region are shown in the bottom panel. Filled colors indicate the linkage disequilibrium r-square values. The y axis on the right-hand side indicates the recombination fractions which is shown by blue curves.
