# Supplementary material for: Genome-wide association study identifies 16 genomic regions associated with circulating cytokines at birth
Source: PLoS Genet. 2020 Nov 23;16(11):e1009163. doi: 10.1371/journal.pgen.1009163 (PMC7721185; doi:10.1371/journal.pgen.1009163)
Supplement: S31 Fig — (PDF) [file pgen.1009163.s042.pdf]

S31 Fig. Polygenic score prediction for discovery sample based on Ahola-Olli et al[1]

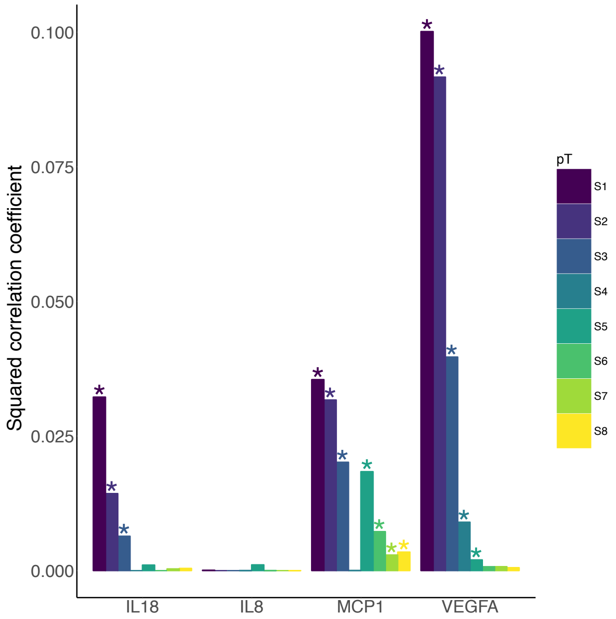

The squared Pearson's correlation coefficients between polygenic scores and measured protein levels in the discovery sample are stratified by different p value thresholds (pT) of association in the discovery sample (S1, P<1x10-6; S2, P <1x10-5; S3,1x10-4; S4, P <0.001; S5, P < 0.01; S6, P < 0.1; S7, P <0.5; S8, P <1.0). The effect sizes for computing polygenic scores are derived from Ahola-Olli *et al.[1]* Stars indicate significant correlation after Bonferoni correction.

1. Ahola-Olli AV, Wurtz P, Havulinna AS, Aalto K, Pitkanen N, Lehtimaki T, et al. Genome-wide Association Study Identifies 27 Loci Influencing Concentrations of Circulating Cytokines and Growth Factors. American journal of human genetics. 2017;100(1):40-50. Epub 2016/12/19. doi: 10.1016/j.ajhg.2016.11.007. PubMed PMID: 27989323; PubMed Central PMCID: PMCPMC5223028.
