# Supplementary material for: Genome-wide association study identifies 16 genomic regions associated with circulating cytokines at birth
Source: PLoS Genet. 2020 Nov 23;16(11):e1009163. doi: 10.1371/journal.pgen.1009163 (PMC7721185; doi:10.1371/journal.pgen.1009163)
Supplement: S33 Fig — (PDF) [file pgen.1009163.s044.pdf]

S33 Fig. Pearson's correlation among inflammation markers.

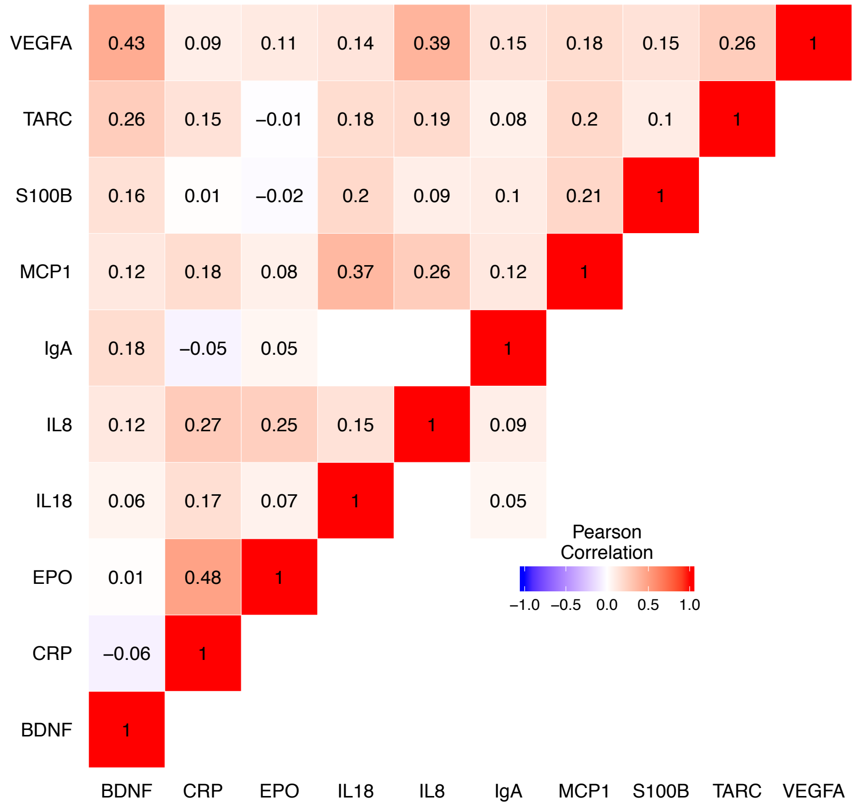

The Pearson correlation coefficients between each pair of age-corrected measures of serum protein levels are shown. Colors indicate the magnitude and direction of the correlation, positive (red) and negative (blue);
