# Supplementary material for: Genome-wide association study identifies 16 genomic regions associated with circulating cytokines at birth
Source: PLoS Genet. 2020 Nov 23;16(11):e1009163. doi: 10.1371/journal.pgen.1009163 (PMC7721185; doi:10.1371/journal.pgen.1009163)
Supplement: S34 Fig — (PDF) [file pgen.1009163.s045.pdf]

S34 Fig. Enrichment of genetic variants with DNAse hypersensitive sites (DHS)

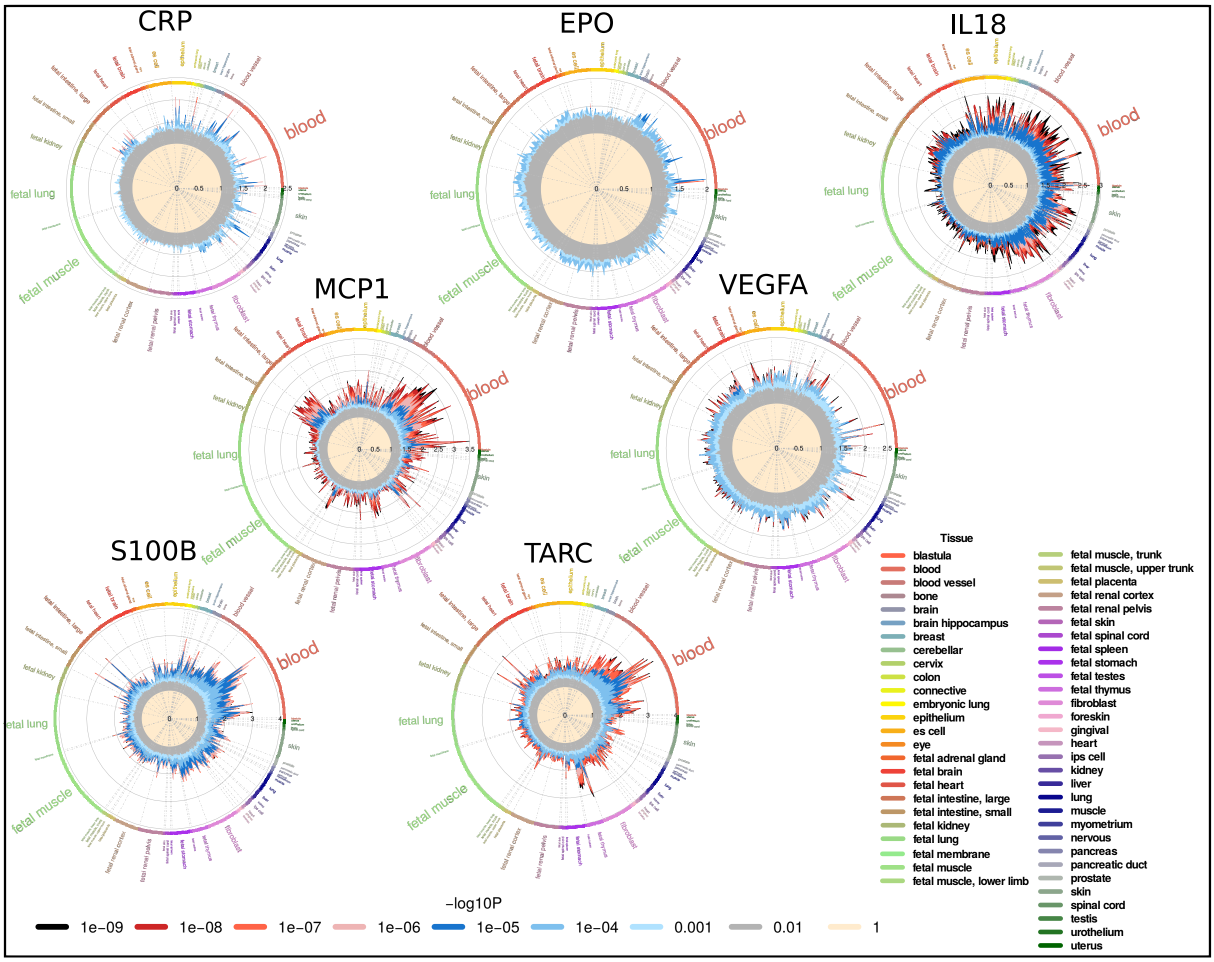

Folds of enrichment are shown by radial spikes for strata of association p values ($<1\times10^{-9}$, 1×10^{-8}, 1×10^{-7}, 1×10^{-6}, 1×10^{-5}, 1×10^{-4}, 1×10^{-3}, 1×10^{-2} and 1.0) for all ENCODE[1] and Roadmap Epigenomics[2] DHS cell lines, sorted by tissue on the outer circle. There is no significant enrichment observed after multiple testing correction. MCP1, TARC and S100B show enrichment in blood.

1. Kavanagh D, Dwyer S, O'Donovan M, Owen M. The ENCODE project: implications for psychiatric genetics. Molecular psychiatry. 2013;18(5):540-2.

2. Kundaje A, Meuleman W, Ernst J, Bilenky M, Yen A, Heravi-Moussavi A, et al. Integrative analysis of 111 reference human epigenomes. Nature. 2015;518(7539):317-30. Epub 2015/02/20. doi: 10.1038/nature14248. PubMed PMID: 25693563; PubMed Central PMCID: PMCPMC4530010.
