# Supplementary material for: Genome-wide association study identifies 16 genomic regions associated with circulating cytokines at birth
Source: PLoS Genet. 2020 Nov 23;16(11):e1009163. doi: 10.1371/journal.pgen.1009163 (PMC7721185; doi:10.1371/journal.pgen.1009163)
Supplement: S35 Fig — (PDF) [file pgen.1009163.s046.pdf]

S35 Fig. Enrichment of genetic variants with histone modification

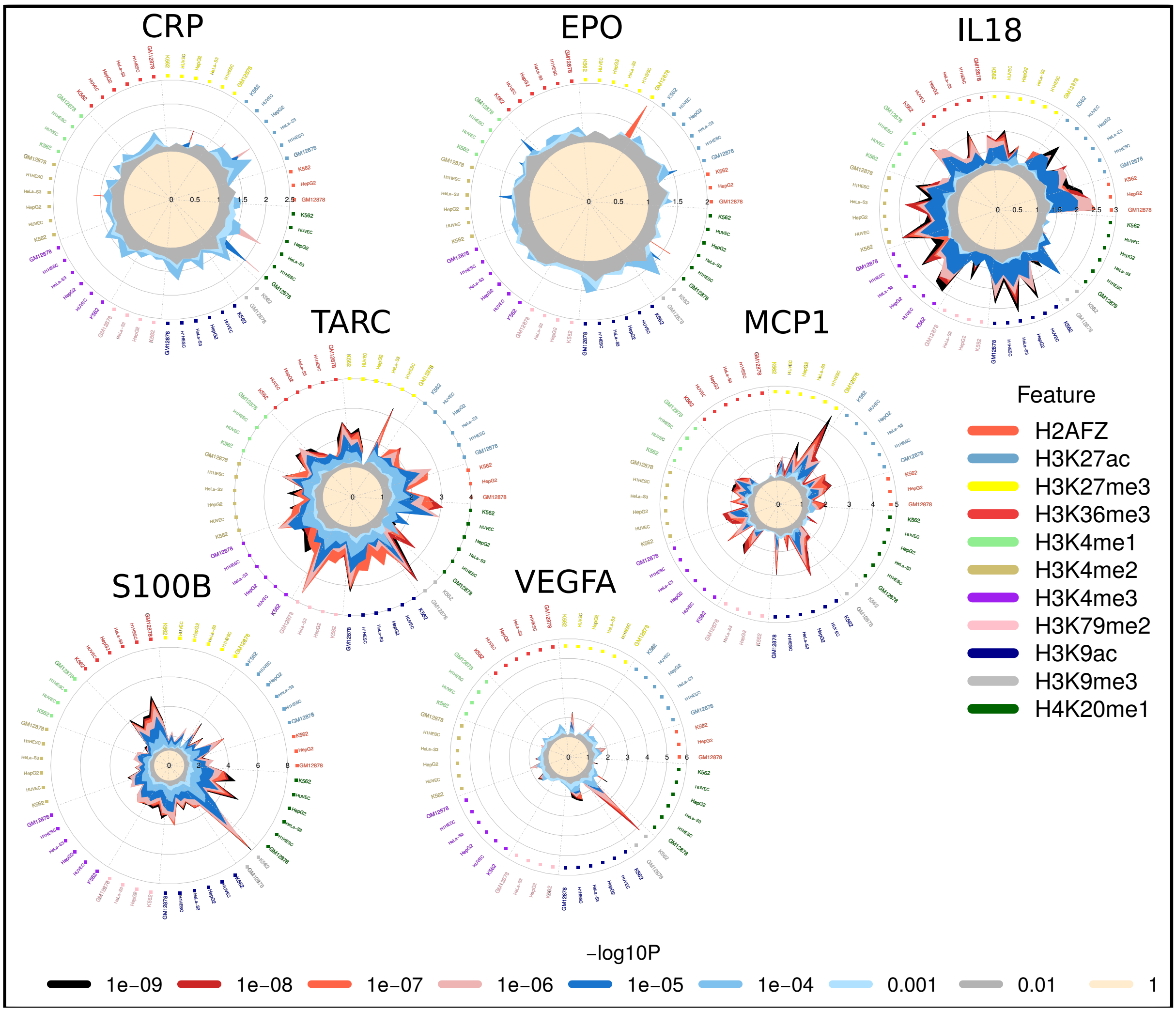

Folds of enrichment are shown by radial spikes for strata of association p values ($<1x10^{-9}$, $1x10^{-8}$, $1x10^{-7}$, $1x10^{-6}$, $1x10^{-5}$, $1x10^{-4}$, $1x10^{-3}$, $1x10^{-2}$ and 1.0) for all histone marks in ENCODE[1] and Roadmap Epigenomics[2] cell lines, sorted by cell lines on the outer circle. There is no significant enrichment observed after multiple testing.

1. Kavanagh D, Dwyer S, O'Donovan M, Owen M. The ENCODE project: implications for psychiatric genetics. Molecular psychiatry. 2013;18(5):540-2.

2. Kundaje A, Meuleman W, Ernst J, Bilenky M, Yen A, Heravi-Moussavi A, et al. Integrative analysis of 111 reference human epigenomes. Nature. 2015;518(7539):317-30. Epub 2015/02/20. doi: 10.1038/nature14248. PubMed PMID: 25693563; PubMed Central PMCID: PMCPMC4530010.
