# Supplementary material for: Genome-wide association study identifies 16 genomic regions associated with circulating cytokines at birth
Source: PLoS Genet. 2020 Nov 23;16(11):e1009163. doi: 10.1371/journal.pgen.1009163 (PMC7721185; doi:10.1371/journal.pgen.1009163)
Supplement: S37 Fig — (PDF) [file pgen.1009163.s048.pdf]

S1K1 Fig. Power analysis of discovery and replication samples.

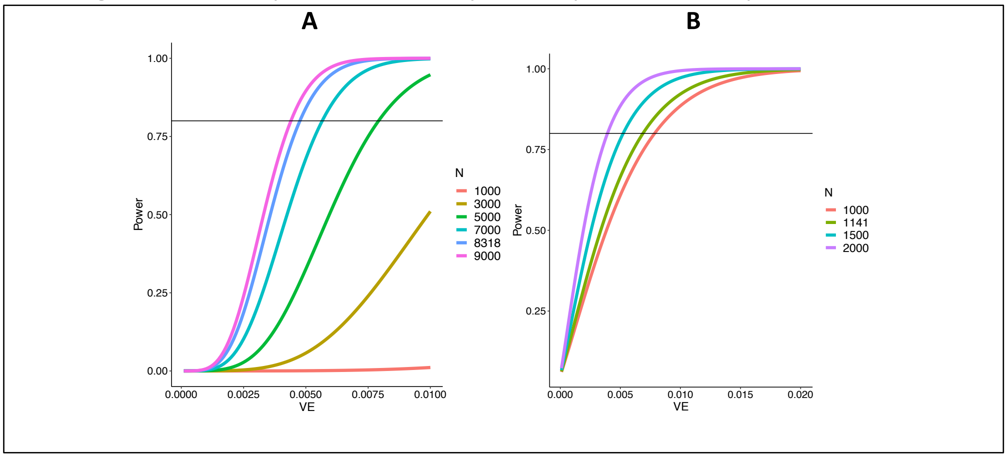

Power analysis for discovery analysis(A) and replication analysis (B). The statistical power (y axis, Power) for identifying significant SNPs (p<=5x10-8 for A and 0.05 for B) was plotted versus proportions of phenotype variance explained (X axis, VE). Different sample sizes were indicated by colors. The horizontal black lines indicate 0.8 power.
