# Supplementary material for: Genome-wide association study identifies 16 genomic regions associated with circulating cytokines at birth
Source: PLoS Genet. 2020 Nov 23;16(11):e1009163. doi: 10.1371/journal.pgen.1009163 (PMC7721185; doi:10.1371/journal.pgen.1009163)
Supplement: Attachment — Submitted filename: Wang_et_al_response.docx [file pgen.1009163.s049.docx]

We thank the editors and reviewers for their helpful and constructive comments. We have addressed all comments below on a point-by-point basis. We believe that the manuscript has much improved as a result, and we hope that our response is satisfactory.

This is an original and interesting manuscript. The reviewers have highlighted the need for additional clarification and technical details. It would also be of interest to elaborate on the potential overlap in genetic architecture between adult and neonatal genetic variation associated with cytokines, perhaps through the application of LD score regression.
*Response: We thank the editor for the positive comments. We agree that estimation of genetic correlations between our data and previously published studies will further strengthen our conclusions. However, the summary statistics-based models, such as LD score regression, depend heavily on the heritability of the proteins and the sample sizes of the GWAS. The author of LD score regression recommends using GWAS with at least 10,000 subjects for complex human diseases/traits. We have tried to apply LD score regression to the four cytokines (IL8, IL18, MCP1 and VEGFA) that were assayed both in our study and in Ahola-Olli et al[1]. We found that only MCP1 showed significant genetic correlation between the two studies (p=0.003, rg=1.06, se=0.37). Possible reasons may be that the Ahola-Olli study included fewer samples than ours and their assay was performed at different ages for the subjects. The estimated heritabilities from the Ahola-Olli study were lower than those estimated from our study:*

*IL8: N=3526, h2=-0.04, se=0.06 vs. N=8318, h2=0.07, se=0.05;*

*IL18: N=3636, h2=0.02, se=0.08 vs. N=8318 h2=0.35, se=0.18;*

*MCP1: N=8293, h2=0.167, se=0.08 vs. N=8318, h2=0.174, se=0.074; and,*

*VEGFA: N=7118, h2=0.05, se=0.06 vs. N=8318, h2=0.15, se=0.07.*

*Thus, our study has a larger power in discovering genetic loci associated with the level of cytokines.*

*Response to Reviewer's Questions:*

**Comments to the Authors:
Please note here if the review is uploaded as an attachment.**

Reviewer #1: This study by Wang et al. performs a genome wide association studies examining ten cytokines extracted from neonatal blood birth spots. The study is well performed and accompanied by SNP heritability, fine-mapping and through polygenic risk score analysis compares the genetic architecture in adults to levels of cytokines in newborns.
*Response: Thank you for your positive comments.*

Major comments:

A power calculation has not been presented. This could be performed based on the previous cytokine GWAS by Ahola-Olli et al and indicated whether the numbers are sufficient. This could then indicate the numbers needed for the replication sample. Additionally, is cytokine data only available 12,000 individuals within the Danish iPSYCH?
*Response: Thank you for pointing this out. We have performed power analysis for both discovery and replication analysis using a quantitative genetic model that assumes a linear regression model of the levels of cytokines on the dosage of SNP alleles, i.e., 0, 1, 2 copies pf the reference allele (*<https://genome.sph.umich.edu/wiki/Power_Calculations:_Quantitative_Traits>)*. For discovery study we assume a significant level of alpha=5×10^-8^ and for the replication study we assumed alpha=0.05 (Fig. 1).*

**A**	**B**

Fig 1. Power analysis for discovery analysis(**A**) and replication analysis (**B**). The statistical power (y axis, Power) for identifying significant SNPs(p<=5x10-8 for **A** and 0.05 for **B**) was ploted versus propotion of phenotype variance explained (X axis, VE). Different sample sizes were indicated by colors.The horizontal black line indicate 0.8 power.

*We show our discovery sample has >0.8 power to identify SNPs that can explain as much as 3×10^-3^ of the phenotype variance. Of the 20 loci we discovered, 14 have power > 0.8. For the smaller replication sample, only SNPs that have explained as much as 6×10^-3^ phenotypic variance can be identified at alpha<= 0.05 with a power > 0.8. This means of the ten loci we tried to replicate only three have this power, i.e., two for S100B and one for IL18. However, the remaining loci have been reported by previous studies. In addition, both the discovery and the replication samples are large enough to discover and replicate loci that associated with the level of S100B, which are the first time been studied.*

*And, yes, there are 12,000 subjects of iPSYCH that have cytokines measured.*

*Edit: We have added the results of the power analysis to the supplementary Figure S37.*

The split between the discovery and replication samples needs to be explained. This appears not to be random due to the loss of almost half the individuals in the replication sample after removing the non-Danish individuals compared to only a fifth in the discovery. Equally, only half of the cytokines are present in the replication sample.
The abstract and discussion is then misleading in terms of number of individuals and number of cytokines included in this study.

*Response: The discovery and replication samples were selected and examined at different time points using different cytokine assay technology. The replication sample was selected only from the second wave of the 23 waves of iPSYCH, and assayed earlier than the discovery sample using the Luminex techonology [2]. The discovery sample was randomly selected from all the remaining samples of iPSYCH at a later stage and assayed using a different technology [3]. Therefore, in this study we did not design a proper replication study but rather tested the robustness of our findings by existing data using different samples generated by different cytokine assay methodologies. The disparities in the proportion of non-Danish sample may reflect the difference between the second wave and the other waves. However, we applied the same protocol for defining Danish vs. non-Danish for both the replication and the discovery samples so this difference would not affect our findings. We essentially chose the larger of the two samples (assayed via different methodologies) as our discovery sample to increase power.*

*We have updated the actual numbers (i.e., 9,459) based on which samples our results were generated with in the Abstract and Author Summary.*

There are analysis and figures in the supplementary material that are not referred to in the text at all (S33-38). These analyses would be very interesting to discuss in the results.

*Response: Thank you for spotting this. We have updated our Results section to include the references to Figure S34, S36 and S37. We have now removed Figures S33 and S35 from supplementary information. These two figures showed our preliminary analysis on the relationships between cytokine levels and psychiatric and autoimmune disorders. We agree with the reviewer that such an investigation is a very interesting topic and worth discussion. However, we were aware of that our sample includes psychiatric patients (Table S1) and our sample overlaps with several public GWAS of psychiatric diseases (SCZ and BIP). Thus, we believe that a future study that properly deals with these two issues should be performed.*

*We have added the following text to our Result section.*

“As expected, we observed both positive and negative correlations of the measured marker levels, correlation coefficients range from -0.06 to 0.43, but positive correlation is the majority (**Figure S33**). This suggests a complex regulation mechanism for immune responses.

We also studied if these cytokine associated SNPs were enriched in DNAse hypertensive sites, histone modification and Chromatin State. However, after correcting multiple testing no significant enrichment was discovered (**Figure S34** -**S36**).”

The introduction and discussion should be expanded. They could include for example:
Introduction: why choice of those particular markers; what the markers are; merits of performing GWAS; etc. Discussion: not a proper replication; clinical relevance of knowing an individual’s genetic predisposition to cytokines; PGRSs only correlating for certain cytokines; etc.
*Response: We agree with the reviewer. We have considerably expanded the introduction and the discussion.*

In the inflammation marker level measurements, the samples with concentrations falling below the lowest concentration within the working range should be excluded rather than being assigned to that value.

*Response: Thank you for pointing this out. How to handle the sample below the detection limits properly is a difficult topic of research in chemometrics. And, the choice of method depends on specific sample. We are aware of that there are several advanced models to handle it such as methods described in Dannis R. Helsel [4]. However, in our sample, there were less than 50 samples on average having a value below the detection limits. We have compared the results of analyses excluding those samples with ones assigning the detection limits to those samples. As expected, there was no qualitative changes in the results. Thus, we present the latter in our manuscript, which has a larger sample size.*

The polygenic risk scores need more of an explanation: it is unclear why only specific ones were generated. I’m not convinced presenting it in terms of pvalue thresholds adds to the conclusions.

*Response: The cytokines that have polygenic risk score results are those that were measured both in our discovery sample and the replication sample, or have been studied by Ahola-Olli et al [1]. We presented the PGRS results by stratifying SNPs by their association p values. The goal was to make an easier comparison in the genetic architecture of cytokines with those of complex traits, e.g. schizophrenia[5], human height [6]. The strength of associations between PGRS and complex traits typically increases as the p value thresholds increase until reaching a plateau. However, for the cytokines in our study the strongest associations were obtained with the genome wide significant SNPs. We agree that this is only an descriptive analysis but it does provide some insights.*

There needs to be more explanation about the conclusion of cis regulatory mechanisms from the proportion of SNP heritability from each chromosome and the extreme p values.
*Response: We inferred our conclusion of cis-regulation by counting the number of genome-wide significant SNPs near the coding genes of the corresponding cytokines as shown in Table 1 in our manuscript. Then, we used the partition of heritability into each chromosome to confirm our results. The chromosomes containing the coding genes of the respective cytokines always account for most of the heritability. Thus, we concluded that cis-action may be the rule for cytokine regulations. In addition, we estimated the proportion of SNP-heritabilities accounted for by the coding regions for S100B, IL18 and CRP using LD score regression. The coding regions were defined as from 500kb up of start site and 500kb down of end site of the coding gene for each cytokine. Briefly, we first estimated the heritabilities accounted for by whole genome SNPs, then we re-estimated it excluding the corresponding coding region. The differences were taken as the portion of heritabilities accounted for by the coding region. Since LD score regression gives large standard errors with our sample, we estimated the range of the proportion of heritabilities accounted for by coding regions by +/- one standard error of the two estimates, and then subtract it from 1. The results for S100B is 0.48 – 0.82; For CRP, it is 0.22-0.24; and for IL18, it is 0.24-0.54. This results further support our cis-regulation hypotheses.*

*Several possible explanations for the extreme p values observed. First, our sample sizes are largest by the time of submission. This can increase the statistical power of variants discovery (see power curve above). Second, our cytokine assay was performed on neonate dried blood spots, i.e., 5-7 days after birth. This may remove large part of environmental influences on the cytokine levels. These strengths were reflected by the higher SNP-heritability estimates as shown in figure 1 compared with the Ahola-Olli study. In addition, SNPs that show extreme association p values are biologically plausible, e.g., most of them are near the corresponding coding genes.*

Please double check the colour scheme is suitable for colour blind people. *Response: Thank you for making this point. We used the viridis color scheme (*<https://github.com/sjmgarnier/viridis>) *for our figures. This scheme has been shown to be friendly with readers with the most common form of color-blindness. Indeed, the first author of this study is red-green colorblind thus we believe that our color scheme is appropriate.*

Results:
The individual figures from the supplementary need to be referenced in the results rather than just ‘Appendix S1’.
*Response: Thank you. We have updated our reference to supplementary figures.*

Line 110 and 111: should be SNP heritabilities.

*Response: We have corrected it in the revised manuscript.*

Line 113: How much of the SNP heritability stems from the coding genes? Because if that explains the SNP heritability then you can’t conclude that there are strong cis-regulatory mechanisms.

*Response: Thanks for pointing this out. We further confirmed our claim of cis-regulated cytokines (S100B, IL18 and CRP) by estimates the SNP-heritability accounted for by the coding regions (see above).*

Line 114: ‘analyses suggest’ is surplus.

*Response: Thank you. We have corrected it in the revised manuscript.*

Line 140: ‘clumped’ needs a better explanation

*Response: Thank you. We have defined how we performed clumping at the Method section as per below:*

“***Associated regions and genes***

Association results were ‘clumped’ using PLINK based on the linkage disequilibrium structure of the 1000 Genomes projects phase 3 EUR dataset, with parameters *–clump-p1 5e-9 –clump-2 1e-6 –clump-r2 0.1.* Five hundred kilo-base (kb) were used as inter-region distance threshold. Genes whose genomic coordinates located within the boundaries of each region were assigned to the corresponding region. SNPs with the smallest association p values were taken as the leading SNP for the corresponding region.”

Methods:
The discovery and replication p value needs to be justified.

*Response: For discovery analysis we used p < 5x10^-9^, which is 5x10^-8^/10 cytokines, as our genome-wide significant threshold. And, for replication, we took a liberal threshold p <= 0.05 as most loci have been reported previously.*

Is there a justification for only including 6 principal components?

*Response: We selected PCs to include by first performing a linear regression analysis of the levels of each cytokine on each PC. Since the PCs were derived from genotypes only without regards to cytokines and were ranked from the one accounting for the most variations to the least. We employed the first 6 PCs following regression analyses testing each PC and each cytokine until we reached a PC which was not associated (P>0.05) with any of the 10 cytokines. As a result, we included six PC in all cytokine analysis. We believe our strategy is more conservative as some of the PC 1-6 were not significantly associated with all cytokines.*

Is there a reason that the chromosomes X and Y were excluded?
*Response: In this study we did not focus on sex chromosomes. However, this is an interesting idea for future studies.*

Line 269: ‘BOLT-RMEL’ should be ‘BOLT-REML’

*Response: We have corrected it in the revised manuscript.*


Figures:
Figure 1b needs better labelling – it is currently very confusing to understand.

*Response: We agree that Figure 1b may give readers the impression that each cytokine has a similar SNP-heritability. It should be understood in relation to Figure 1a which shows SNP heritability for each cytokine. To make it easier we updated our Figure1b by adding the numeric estimates of SNP heritabilities to the figure.*

*Edit: We have updated Figure 1 to this new form.*

The variation of circulating marker levels captured by **a.** all the genotyped SNP; **b.** SNPs on each autosome and **c.** polygenic scores computed from independent sample are shown. Cytokine SNP-heritabilities were shown in **a** by point estimates and standard errors. These point estimates were also shown in parentheses following the cytokine names on **b**. The Pearson’s correlation coefficients between polygenic scores and measured protein levels in the discovery sample are stratified by different p value thresholds (pT) of association in the discovery sample (S1, P<1x10^-6^; S2, P <1x10^-5^; S3,1x10^-4^; S4, P <0.001; S5, P < 0.01; S6, P < 0.1; S7, P <0.5; S8, P <1.0). The effect sizes used to compute polygenic scores are derived from Ahola-Olli *et al.*[1].

Reviewer #2: This study intended to find genetic contributors for ten inflammation markers by performing GWAS of two samples. They found and replicated 16 associated genomic regions, of which four are novel. Further, they estimated SNP-based heritability ranging from 0 for EPO up to 73% for S100B. Finally, the authors mapped these associated variants to enhancer elements, suggesting a possible transcriptional effect of genomic variants on the inflammatory markers. Overall, this is a well-designed and conducted study with many strengths and merits. However, the major concern of this paper is about its writing. In many parts, description is over simplified, which made this paper hard to follow. It would be important and essential for the authors to expand almost all parts of the paper.

*Response: We thank the review for the positive comments. We have expanded our manuscript to include more information. See the response to reviewer 1 and our revised manuscript.*

Reviewer #3: The manuscript is well-written and provides important insights into genetics of inflammatory biomarkers. The results contain interesting genetic instruments for use in future Mendelian randomization studies. I have few questions related to manuscript.

1) The biomarkers were measured from dried blood spots. It is well known that for example heparin releases cytokines from receptors, such as ACKR1 (DARC), which serves as cytokine reservoir on red blood cell surface. This release induced by heparin might mask some genetic signals which would be detected if quantification would have been done by using non-heparin treated blood samples. Is there any previous data on how cytokine measures done from dried blood correlates with measures done from plasma or serum?

*Response: We have developed and validated the procedure of cytokine assay using dry blood spots, serum and plasma in our previous studies[2,7]. Skogstrand et al[2] has shown that dried blood spots are actually a much more precise way of drawing blood for cytokine measurement compared to plasma and serum, as leukocytes in all samples are “stopped” at the same time. In plasma and serum, different times and temperatures between sample draw and centrifugation, will contribute to high variation in cytokine levels as the leukocytes will continue to secrete cytokines in the sample [2]. This will not happen in dried blood spots. Further, there are no additives like anticoagulants in dried blood spots. Cytokine concentrations measured in plasma, serum and dried blood spots cannot be directly compared due to above-mentioned factors + differences in the matrixes’ influences on the assays like plasma-binding proteins etc. There is, however, a correlation between high and low samples.*

2) Could you explain why you calculated SNP heritability explained by each chromosome by excluding the pertinent chromosome from SNP heritability estimation and then subtracting the obtained SNP heritability from total SNP heritability explained by all autosomes instead of just calculating SNP heritability for one chromosome at a time?
*Response: We used the linear mixed-effect models (the programme BOLT[8]) for estimating SNP heritabilities. These models rely on the estimates of the realized genetic relationship between individuals using SNPs. Thus, the higher of the coverage of the genome by independent SNPs used the less biased the estimates of the relationship between subjects. For this reason, we used the whole genome SNPs after removing correlated SNPs for estimating heritabilities accounted for by SNPs, and then subtracted from it the estimates without including each chromosome to partition heritabilities to each chromosome.*

3) According to Skogstrand et al. inter-assay variability for S100B was over 13%. Were cytokines assayed independently from genotyping batches?
*Response: The assay of S100B showed a relatively larger variation across assay compared with other cytokines. However, in the present study, both the discovery and replication samples are statistically powered to identify the variants that we reported. In addition, the most significant loci identified for S100B are around the coding gene for this protein, which indicates that such technical noise has little effect on our results. The replication sample was randomly selected from the second wave of the iPSYCH sample. And, the discovery sample was randomly selected from the remaining subjects of the 23 waves of iPSYCH. The cytokine assays were independent of the genotypes.*

4) S100B quantification was done with in-house developed platform. Do you know what this approach actually measures? Can capture antibody block the binding of detection antibody?
*Response: S100B was set up as a sandwich immunoassay on the Meso-Scale platform. Capture antibodies are bound specifically to the wells, following incubation with the samples where S100B will be bound to the capture antibodies. At the last step, detection antibodies are added, and these will bind to another epitope on S100B. The capture antibody used (Sigma-Aldrich S25302), is monoclonal and binds specifically to an epitope located on the β-chain, while the detection antibody (Agilent (former DAKO) Z0311), are polyclonal, thus are able to bind to more than one epitope on S100B. The capture antibody will therefore not block the binding of the detection antibody. Assay validation included tests for cross-reactivity, and the only antigen detected was S100B. As for most immunoassays, we cannot be sure if we only detect the free form or also the plasma bound form of the protein.*

5) The Supplementary tables don't have any foot notes or explanation where the data originated from. Therefore, it is hard to track what data they actually contain. Could you describe these little more specifically on each spreadsheet?
*Response: We apologize for the not-so-clear presentation. We have updated our description for supplementary spreadsheets.*

*We have added the following description to Tables S2-S8:*

“Additional annotations for SNPs associated with *XXX* levels.

Sheet 1: Panel FineMapping: Fine mapping of significant associations using FINEMAPFINEMAP (http://www.christianbenner.com/) assuming up to three causal variants per locus. The Following columns are presented: SNP: SNP rs number; CHR: Chromosome number; POS: genomic position of the SNP; A1: The effective allele; A2: The other allele; : Association p value; Zscore: Association z-score; PIP: Posterior inclusion probability; LBF: log10 Bayesian factor of the causal set.

Panel Ensembl VEP: Annotations of SNPs by the Ensembl-VEP online application (http://grch37.ensembl.org/Homo_sapiens/Tools/VEP). The results were based on Assembly GRCh37.p13. The transcript database GENCODE transcripts and basic transcripts were used. For the Predictions SIFT, CADD, polyPhen, and LoFtool were used. The default settings were used for Additional annotations, Filtering options, and Advanced Options. The following columns are presented: Gene: Gene symbol of the annotated Gene; position: Position of the SNP relative to the annotated gene; Distance: Genomic distance from the SNP to the annotated gene; Impact: predicted impact of the SNP to the expression of the annotated gene; LoFtool: Predicted impact of the SNP by the LoF tool; CADD_PHRED: Predicted impact of the SNP by CADD.

Panel HaploReg v4.1: Annotations of SNPs by the HaploReg v4.1 webtool (https://pubs.broadinstitute.org/mammals/haploreg/haploreg.php). The EUR sub-pouplation from 1000G phase 1 was used for LD calculation and 0.8 was used as LD r2 threshold. In addition, both was selected for the Mammalian conservation algorithm, ChromHMM (Core 15 state model) , ChromHMM (25-state model using 12 imputation), H3K4me1/H3K4me3 peaks, H3K27ac/H3K9ac peaks for Source for epigenomes was selected separately, and GENCODE genes for Show position relative to. The following columns are presented: Epigenome IDs: Epigenome identifiers from the database; Epigenome groups: group of the tissue used for the epigenomic data generation; Description: short description for the Epigenome dataset; Chromatin stats (Core 15-state model), Chromatin states (25-model using 12 imputed marks), H3K27ac, H3K4me1, H3K4me3, and DNase, results for different Source for epigenomes.

Sheet 2:

SNP annotations by the VarElect-Enhancer program from the GenCards database v4.5 (https://www.genecards.org/). The following columns presented: LD_R2: LD r2 with the leading SNP; SNP: SNP rs number; BP_orig: Genomic position of the SNP; CHR: Chromosome number; A1:The Effective allele; A2: The other allele; FRQ: Frequency of A1 in our sample; INFO: Imputation INFO score for the SNP; BETA: Effect size ofA1 association with the cytokine levels; SE: Standard error of BETA; P: Association p value; GHid: Gene enhancer ID from the GeneCards database; enhScore: Enhancer score; isElite: whether the enhancer is double-elit (yes: 1 and no: 0), i.e., enhancer-gene connections for which both the enhancer itself and the connection to the gene are supported by at least two sources or methods, respectively.

Sheet 3:

Information about the enhancers in Sheet 2 obtained from the GeneCards database v4.5. The following columns are presented: GHid: Enhancer ID; symbol: Symbol of the gene regulated by the enhancer; eqtl_score: eQTL annotation score of the enhancer; erna_score: eRNA annotation score for the enhancer; chic_score: Capture Hi-C annotation score of the enhancer; expression_score: Transcription factor correlation with the enhancer; enhancer_gene_distance: Genomic distance from the enhancer to the gene; distance_score: Annotation score of the distance; combined_score: Combined annotation score for the enhancer; is_elite: : whether the enhancer is double-elit (yes: 1 and no: 0); eqtl_tissue: source of tissue for the eQTL annotation. NA, not available.

Sheet 4:

Tissue source of the enhancers in Sheet 2 and 3. The following columns are presented: GHid: Enhancer ID; source: Database from where the annotation was derived; tissue: Tissue used for the enhancer annotation.

Sheet 5:

Colocalization of SNPs by the Sherlock program (http://sherlock.ucsf.edu/). The results are based on the GTEx v6 data. The following columns are presented: Lead_SNP: Leading SNP of association; LD_r2: LD r2 between the SNP and the leading SNP; SNP: SNP rs number; P: Association p value; CHR: Chromosome number; BP: Genomic position; GENE: Implicated gene symbol; cP: Colocalization p value; LBF: log10 Bayesian factor; eP: eQTL p value; Mode: Mode of regulation; Tiss: Source tissue for the expression data.

--end

6) According to the methods each genotyping wave was imputed separately. Sample size is an important determinant of phasing accuracy and this accuracy decreases rapidly when sample size drops below 1000 which in turn impairs imputation accuracy. What was the sample size in each genotyping wave?

*Response: We agree that imputing larger sample at once can improve imputation accuracy. However, the iPSYCH sample were genotyped separately into 23 waves and each wave has more than 2000 subjects. With this setting we believe that imputation by waves is more appropriate. The imputed genotypes have been carefully compared and quality controlled as described also in our previous studies[9-11] and in the description of the iPSYCH design [12].*

*References*

*1. Ahola-Olli AV, Wurtz P, Havulinna AS, Aalto K, Pitkanen N, et al. (2017) Genome-wide Association Study Identifies 27 Loci Influencing Concentrations of Circulating Cytokines and Growth Factors. Am J Hum Genet 100: 40-50.*

*2. Skogstrand K, Thorsen P, Norgaard-Pedersen B, Schendel DE, Sorensen LC, et al. (2005) Simultaneous measurement of 25 inflammatory markers and neurotrophins in neonatal dried blood spots by immunoassay with xMAP technology. Clin Chem 51: 1854-1866.*

*3. Skogstrand K, Hagen CM, Borbye-Lorenzen N, Christiansen M, Bybjerg-Grauholm J, et al. (2019) Reduced neonatal brain-derived neurotrophic factor is associated with autism spectrum disorders. Transl Psychiatry 9: 252.*

*4. Dennis R H (2004) Nondetects and data analysis: Statistics for censored environmental data: Wiley-Interscience. 268 p.*

*5. Consortium SWGotPG (2014) Biological insights from 108 schizophrenia-associated genetic loci. Nature: 421–427.*

*6. Yengo L, Sidorenko J, Kemper KE, Zheng Z, Wood AR, et al. (2018) Meta-analysis of genome-wide association studies for height and body mass index in ∼700000 individuals of European ancestry. Hum Mol Genet 27: 3641-3649.*

*7. Skogstrand K (2012) Multiplex assays of inflammatory markers, a description of methods and discussion of precautions - Our experience through the last ten years. Methods 56: 204-212.*

*8. Loh PR, Tucker G, Bulik-Sullivan BK, Vilhjalmsson BJ, Finucane HK, et al. (2015) Efficient Bayesian mixed-model analysis increases association power in large cohorts. Nat Genet 47: 284-290.*

*9. Grove J, Ripke S, Als TD, Mattheisen M, Walters RK, et al. (2019) Identification of common genetic risk variants for autism spectrum disorder. Nat Genet 51: 431-444.*

*10. Demontis D, Walters RK, Martin J, Mattheisen M, Als TD, et al. (2019) Discovery of the first genome-wide significant risk loci for attention deficit/hyperactivity disorder. Nat Genet 51: 63-75.*

*11. Nudel R, Wang Y, Appadurai V, Schork AJ, Buil A, et al. (2019) A large-scale genomic investigation of susceptibility to infection and its association with mental disorders in the Danish population. Translational Psychiatry 9: 283.*

*12. Pedersen CB, Bybjerg-Grauholm J, Pedersen MG, Grove J, Agerbo E, et al. (2017) The iPSYCH2012 case-cohort sample: new directions for unravelling genetic and environmental architectures of severe mental disorders. Mol Psychiatry.*
